# MBD2 mediates Th17 cell differentiation by regulating MINK1 in Th17-dominant asthma

**DOI:** 10.3389/fgene.2022.959059

**Published:** 2022-10-11

**Authors:** Zhifeng Chen, Yulin Shang, Yu Yuan, Yi He, Binaya Wasti, Wentao Duan, Ruoyun Ouyang, Jingsi Jia, Bing Xiao, Dongshan Zhang, Xiufeng Zhang, Jianmin Li, Bolin Chen, Yi Liu, Qingping Zeng, Xiaoying Ji, Libing Ma, Shaokun Liu, Xudong Xiang

**Affiliations:** ^1^ Department of Respiratory Medicine, The Second Xiangya Hospital, Central South University, Changsha, Hunan, China; ^2^ Ophthalmology and Otorhinolaryngology, Zigui County Traditional Chinese Medicine Hospital, Zigui, Hubei, China; ^3^ Department of Emergency, The Second Xiangya Hospital, Central South University, Changsha, Hunan, China; ^4^ Department of Respiratory Medicine, The Second Affiliated Hospital of Hainan Medical University, Haikou, Hainan, China; ^5^ Department of Respiratory and Critical Care Medicine, Hunan Provincial People’s Hospital, Changsha, Hunan, China; ^6^ Department of Respiratory Medicine, Zhuzhou City Central Hospital, Zhuzhou, Hunan, China; ^7^ Department of Respiratory and Critical Care Medicine, Longshan County People’s Hospital, Longshan, Hunan, China; ^8^ Department of Respiratory and Critical Care Medicine, The Affiliated Hospital of Guizhou Medical University, Guiyang, Guizhou, China; ^9^ Department of Respiratory and Critical Care Medicine, The Affiliated Hospital of Guilin Medical University, Guilin, Guangxi, China

**Keywords:** Th17-dominant asthma, misshapen like kinase 1, methyl-cpg binding domain protein 2, t-helper cell type 17 cells, bronchial epithelial cells

## Abstract

**Objectives:** .Asthma is a highly heterogeneous disease, and T-helper cell type 17 (Th17) cells play a pathogenic role in the development of non-T2 severe asthma. Misshapen like kinase 1 (MINK1) is involved in the regulation of Th17 cell differentiation, but its effect on severe asthma remains unclear. Our previous studies showed that methyl-CpG binding domain protein 2 (MBD2) expression was significantly increased in patients with Th17 severe asthma and could regulate Th17 cell differentiation. The aim of this study was to investigate how MBD2 interacts with MINK1 to regulate Th17 cell differentiation in Th17-dominant asthma.

**Materials and methods:** Female C57BL/6 mice and bronchial epithelial cells (BECs) were used to establish mouse and cell models of Th17-dominant asthma, respectively. Flow cytometry was used to detect Th17 cell differentiation, and the level of IL-17 was detected by enzyme-linked immunosorbent assay (ELISA). Western blot and quantitative real-time PCR (qRT-PCR) were used to detect MBD2 and MINK1 expression. To investigate the role of MBD2 and MINK1 in Th17 cell differentiation in Th17-dominant asthma, the MBD2 and MINK1 genes were silenced or overexpressed by small interfering RNA and plasmid transfection.

**Results:** Mouse and BEC models of Th17-dominant asthma were established successfully. The main manifestations were increased neutrophils in BALF, airway hyperresponsiveness (AHR), activated Th17 cell differentiation, and high IL-17 levels. The expression of MBD2 in lung tissues and BECs from the Th17-dominant asthma group was significantly increased, while the corresponding expression of MINK1 was significantly impaired. Through overexpression or silencing of MBD2 and MINK1 genes, we have concluded that MBD2 and MINK1 regulate Th17 cell differentiation and IL-17 release. Interestingly, MBD2 was also found to negatively regulate the expression of MINK1.

**Conclusion:** Our findings have revealed new roles for MBD2 and MINK1, and provide new insights into epigenetic regulation of Th17-dominant asthma, which is dominated by neutrophils and Th17 cells. This study could lead to new therapeutic targets for patients with Th17-dominant asthma.

## 1 Introduction

Asthma is an epidemic and highly heterogeneous chronic inflammatory clinical disease that affects all age groups (Stern et al., 2020). Distinct asthma endotypes are used to describe the inflammatory pathways that participate in the pathogenesis of asthma at the cellular and molecular levels (Kuruvilla et al., 2019). The inflammatory pathways of asthma are driven by multiple immune mechanisms. CD4^+^ T cells differentiate into a variety of subtypes, including T helper type 2 (Th2) cells and T helper type 17 (Th17) cells, both of which play an important role in the immune response (Muhammad Yusoff et al., 2020). Currently, most studies classify asthma into type 2 (T2) and non-T2 endotypes (Kuo et al., 2017). Sputum eosinophil counts have been identified as biomarkers for classic allergic asthma, which is thought to be driven by a Th2-mediated pathway, known as T2 asthma (asthma) (Peters et al., 2014). However, studies have found that almost 50% of asthma cases are primarily infiltrated by neutrophils rather than eosinophils (a non-T2 endotype) and respond poorly to glucocorticoids. This pathway is driven mainly by Th17 cells, known as Th17-dominant asthma (or neutrophil-dominant asthma) (Douwes et al., 2002; Manni et al., 2016). Th17 cells recruit neutrophils into the airway by secreting the cytokine interleukin-17 (IL-17), which is less susceptible to glucocorticoid inhibition than IL-4 and IL-5 produced by Th2 cells (Newcomb and Peebles, 2013).

Asthma can be triggered by allergies, house dust mites (HDM), oxidative stress, smoking and infections. These risk factors induce an immune response to the initial initiation of T cells by antigen presenting cells (APCs), mainly dendritic cells and bronchial epithelial cells (BECs) (Liu et al., 2020). Several studies have found that BECs can present antigen to T cells and promote the antigen presentation process and subsequent T cell proliferation and differentiation under the action of certain genes, playing a key role in the sensitization and pathogenesis of asthma (Lambrecht and Hammad, 2012; Qu et al., 2013; Lee et al., 2017; Liu et al., 2018).

The germinal center kinase family regulates a variety of cellular processes, including cell growth and differentiation, gene transcription, and immune and stress responses, through the mitogen-activated protein kinase pathways (Fu et al., 1999; Yin et al., 2012). As a member of the germinal center kinase family, misshapen like kinase 1 (MINK1), is a serine-threonine kinase (Dan et al., 2000). MINK1 negatively regulates Th17 differentiation through direct phosphorylation of SMAD2 at T324 residues (Fu et al., 2017). In addition, in animal models of experimental autoimmune encephalomyelitis (EAE), MINK1 deficiency increases IL-17 levels, promoting Th17-dependent inflammation and exacerbating the severity of EAE (Fu et al., 2017). Therefore, we hypothesized that MINK1 is involved in asthma by regulating Th17 cell differentiation and may be a therapeutic target for Th17-dominant asthma.

In addition to genetics, environmental influences also play a role in asthma development, which may be regulated by epigenetic mechanisms (Reese et al., 2019). The epigenetic regulatory mechanisms of asthma include DNA methylation, and the methyl-CpG binding domain (MBD) family of proteins play a “reader” and regulatory role as a key bridge in the process of DNA methylation, and MBD2 is a member of the family (Horsburgh et al., 2015; Shen et al., 2020). MBD2 has been reported to be involved in the pathogenesis of experimental colitis, rheumatoid arthritis, lupus nephritis and other immune diseases (Liu et al., 2011; Zhang et al., 2017; Jones et al., 2020). Zhong et al. found that MBD2 deletion leads to failure to read methylation information, which disrupts T-bet/Hlx homeostasis and leads to reduced differentiation of Th17 cells, thereby playing a protective role in EAE (Zhong et al., 2014). Previous studies have shown that MBD2 is involved in neutrophil-dominant asthma and positively regulates Th17 cell differentiation (Jia et al., 2017; Sun et al., 2018). At present, Th17-mediated asthma is more likely to develop into severe asthma due to poor response to glucocorticoids, and MBD2 may also have a potential therapeutic effect on severe asthma.

A previous study showed that MBD2 was significantly increased in patients with Th17 severe asthma compared to healthy controls and patients with asthma (Chen et al., 2021). In this study, we aimed to determine the role of MBD2 and MINK1 in Th17-dominant asthma. We induced Th17-dominant asthma mouse and BECs models and found that MBD2 was significantly increased *in vivo* and *in vitro*, while MINK1 was decreased, and there was a reverse expression between MBD2 and MINK1. In addition, we also investigated the effect of MBD2 intervention on MINK1 expression and Th17 cell differentiation and found that MBD2 deletion promoted MINK1 expression and inhibited Th17 cell differentiation and IL-17 release. Taken together, these data suggested that MBD2 might promote Th17 cell differentiation by inhibiting MINK1, thereby exacerbating the severity of asthma.

## 2 Materials and methods

### 2.1 Animal model

Female C57BL/6 mice (6–7 weeks old, 18–20 g) were provided by the animal center of the Second Xiangya Hospital of Central South University (Changsha, China) and maintained under specific pathogen-free conditions. All experimental protocols were approved by the Animal Care and Use Committee of the Second Xiangya Hospital of Central South University and were conducted in accordance with the guiding principles of the institution. The establishment of a Th17-dominant asthma mouse model was consistent with previous reports (Daan de Boer et al., 2013; Jia et al., 2017). Animals in the Th17-dominant asthma group (n = 6/group) were given an intraperitoneal sensitization injection containing 100 μg of ovalbumin (1 mg/ml, OVA, Grade V, Sigma Aldrich), 100 μg of HDM (10 mg/ml, Greer Laboratories, United States ) and 15 μg of lipopolysaccharide (1 mg/ml, LPS, Sigma Aldrich) with 2 mg of aluminum hydroxide (Sigma Aldrich) dissolved in 200 μL of saline on days 0,1 and 2. On days 14, 15, 18, and 19, the animals were challenged with 6% OVA solution atomized for 30 min before 100 μg/10 μL HDM was administered intranasally. The normal control group was sensitized and atomized with saline only, but the injection time, location, dose and atomization time were the same as those in the Th17-dominant asthma group.

The T2 asthma group was sensitized by intraperitoneal injection of 100 μg OVA (1 mg/ml) and 2 mg aluminum hydroxide dissolved in 200 μL saline on days 0–7, followed by atomization with 6% OVA solution on days 14–20 for 30 min (Hoffman et al., 2013; Ni et al., 2018). Mice were sacrificed on day 21 for analysis.

### 2.2 Assessment of airway hyperresponsiveness (AHR)

Methacholine (Mch)-induced airway resistance was measured with direct plethysmography (Buxco Electronics, RC System, Wilmington, United States ) on day 21 (Locke et al., 2007). First, baseline lung resistance (RL) was measured for 1 min, then the airways of mice were stimulated with 10 μL of aerosolized saline and 10 μL of Mch at increasing doses (0.39 mg/ml, 0.78 mg/ml, 1.56 mg/ml, 3.12 mg/ml), and RL was recorded again.

### 2.3 Bronchoalveolar lavage fluid processing

Bronchoalveolar lavage fluid (BALF) was collected by injecting 0.5 ml of saline into the lungs 3 times through an endotracheal tube (37°C). BALF inflammatory cells were centrifuged and resuspended in cold phosphate-buffered saline (PBS, 1500 rpm, 5 min, 4°C, Eppendorf Centrifuge Configurator, Hamburg, Germany), then the cells were fixed and stained with Wright-Giemsa, and 200 cells were counted and differentially counted under a light microscope using a counting chamber.

### 2.4 Histopathology

The lungs were fixed with 10% formalin *via* the trachea, then removed and stored in 10% formalin. Fixed lung tissues were paraffinized and sectioned (5 μm) for hematoxylin and eosin (H&E) staining and immunohistochemistry [MBD2 antibody (Abcam, Cambridge, United States ), MINK1 antibody (Proteintech, Wuhan, China), eosinophil antibody (anti-ECP, Biorbyt, Cambridge, United Kingdom) and neutrophil antibody (anti-Gr-1, Biolegend, San Diego, United States )]. Select stained sections per group were collected and assessed for MBD2, MINK1, eosinophil, and neutrophil protein expression.

### 2.5 Bronchial epithelial cell isolation and culture

Bronchial epithelial cells (BECs) were isolated using an improved version of the protocol previously reported (Davidson et al., 2000; Cong et al., 2020). Briefly, bronchi were removed from the gross anatomy of mice, and the tracheae were dissected lengthways, washed with PBS, and transferred to minimal essential medium (MEM, 11095–080, Fisher Scientific International) preheated to 37°C containing 0.1 mg/ml DNase and 1.4 mg/ml pronase (Roche Diagnostics). After incubation at 37°C for 1h, the tube containing the tracheae was carefully inverted 12 times to separate the epithelial cells from the airways. One milliliter of sterile fetal bovine serum (FBS, Gibco, Australia) was added to stop enzyme digestion. After that, a 150-mesh cell sieve (Biosharp, BS-100-XBS, China) was used to remove undigested excess tissue. After centrifugation for 5 min at 800 × *g*, the supernatant was discarded and the cells were resuspended with MEM containing 10% FBS. The cells were then inoculated in culture bottles and left standing at 37°C with 5% CO_2_ for 2 h to remove contaminated non-epithelial cells. Subsequently, the culture medium (including suspended cells) was collected, and the supernatant was removed after centrifugation at 800 *g* for 5 min. BECs were cultured with bronchial epithelial growth medium (Procell, CM-M007, China) in a humidified incubator at 37°C with 5% CO_2_.

Generally, studies have shown that 95% of epithelial cells are positive for cytokeratin, an epithelial marker that is not expressed by lymphocytes (Tryphonopoulos et al., 2004). The BECs were centrifuged onto slides and stained with DAPI and cytokeratin-specific monoclonal antibody (pan-Cytokeratin, SantaCruz, sc-8018, United States ).

### 2.6 Cell asthma model and transfection

To induce Th17-dominant asthma, BECs were treated with 100 μg/ml HDM and 100 ng/ml LPS for 24 h. BECs were treated with 100 μg/ml HDM for 24 h to establish T2 asthma or PBS to establish normal controls (Liu et al., 2019).

Small interfering RNA targeting MBD2, MINK1 (siR-MBD2, siR-MINK1), and negative control (siR-NC) were purchased from RiboBo (Guangzhou, China). The MBD2 interfering sequence was 5′-GCA​AGA​TGA​TGC​CTA​GTA​A-3′ and the MINK1 interfering sequence was 5′-GCA​AGT​ACA​AGA​AGC​GAT​T-3′. Mouse MBD2, MINK1 over-expression plasmids (OE-MBD2, OE-MINK1), and negative control (OE-NC) were purchased from HonorGene (Hunan, China). Small interfering RNA and plasmid were transfected in BECs for 48 h using Lipofectamine 3000 (Invitrogen, United States ) according to the manufacturer’s protocols. After 2 days, the cells were treated with 100 μg/ml HDM and 100 ng/ml LPS for 24 h.

### 2.7 CD4^+^ T cell isolation and cocultivation

Mouse spleen CD4^+^ T cells were isolated by magnetic bead separation (130–117-043, Miltenyi Biotec, Germany). The established asthma model and transfected BECs were cocultured with CD4^+^ T cells (TCs) at a ratio of 10:1 (TCs: BECs) for 24 h, respectively, in complete RPMI 1640 culture medium (Gibco, Australia) supplemented with 10% FBS, 1% penicillin and streptomycin, soluble anti-CD28 (1.0 μg/ml, eBioscience), soluble anti-CD3e (0.5 μg/ml, eBioscience), and IL-2 (20 ng/ml, eBioscience). For analysis of T cell subsets, suspended cells were collected 24 h later, and the concentration of IL-4 and IL-17 A was determined by flow cytometry to obtain the ratio of Th2 to Th17 cells. Total protein of BECs was extracted for western blotting.

### 2.8 Flow cytometry

CD4^+^ T cells were stimulated with 2 μL/ml (1 × 10^6^ cells/ml) leukocyte activation cocktail (550583, BD Biosciences, United States ), and cultured at 37°C with 5% CO_2_ for 6 h, then collected for flow cytometry analysis. After a 6-h incubation, cells were stained with a marker of cell viability (Fixable Viability Stain 510 antibody, BD Pharmingen) for 15 min at room temperature in the dark. Then, cells were stained for surface markers with FITC-anti-CD4 antibody (Biolegend) followed by fixation and permeabilization using the Cytofix/Cytoperm Soln Kit (BD Pharmingen) for 30 min at 4°C in the dark. After washing with permeabilization buffer, cells were stained for intracellular markers with APC-anti-IL-17 A (Biolegend) and PE-anti-IL-4 (BD Pharmingen) antibodies in permeabilization buffer for 30 min at 4°C in the dark. Isotype controls were employed in the control group. Flow cytometry was performed, and data were analyzed using FACS CantoⅡ(Becton Dickinson) and FlowJo version X software.

### 2.9 Western blot

After dissection, the right lung lobes were flash frozen for protein analysis. The lungs were crushed and lysed in a radio immunoprecipitation (RIPA) buffer containing 1% protease inhibitors (Beyotime, Shanghai, China). Proteins in cells were prepared in the same buffer. The protein concentration was measured by using the bicinchoninic acid (BCA) assay (Beyotime, Shanghai, China) according to the manufacturer’s instructions. A 30-μg sample of protein was transferred to the membrane after 1–1.5 h of sodium salt dodecyl sulphate-polyacrylamide gel electrophoresis (SDS-PAGE). The membrane was washed with Tris-buffered saline-Tween 20 (TBST) for 5 min and sealed with 5% skim milk powder at room temperature for 1.5 h. After that, the membrane was incubated with appropriately diluted MBD2, GAPDH (Proteintech, Wuhan, China), and MINK1 antibodies at 4°C overnight. The membrane was incubated with the secondary antibody at room temperature for 1 h. Images were obtained using a chemiluminescence gel imaging system, and the band intensities were measured using ImageJ software (National Institutes of Health). The relative protein expression level was calculated as the ratio of the gray value of target bands to that of GAPDH.

### 2.10 Quantitative real-time PCR

Total RNA from lung tissues and BECs was extracted using TRIzol regent (Invitrogen). The first-strand cDNA was synthesized with the PrimeScript RT Reverse transcriptase Reagent Kit (Takara, Japan). Quantitative real-time PCR (qRT-PCR) was performed using the SYBR Premix Ex Taq kit (Takara, Japan) in the CFX96 Real-Time PCR Detection System (Bio-Rad). *β*-actin was used as the internal reference. According to the cycle threshold (Ct) value of the samples, the expression volume of related genes was calculated by the 2^−ΔΔCt^ method. Primers for target genes were generated by Sangon Biotechnology (Shanghai, China). The primer sequences were: MBD2-forward, 5′-AGT​GCT​GGC​AAG​AGC​GA-3′ and MBD2-reverse, 5′-GCC​GGT​CCT​GAA​GTC​AAA-3′;

MINK1-forward, 5′-CCA​CCT​ACT​ATG​GGG​CCT​TTA-3′ and MINK1-reverse, 5′-AGC​ACC​GCA​GAA​CTC​CAT​C-3′; *β*-actin-forward, 5′-GTG​CTA​TGT​TGC​TCT​AGA​CTT​CG-3′ and *β*-actin-reverse, 5′-ATG​CCA​CAG​GAT​TCC​ATA​CC-3′.

### 2.11 Enzyme-linked immunosorbent assay

The levels of IL-17 and IL-4 in mouse serum and cell supernatant were determined by enzyme-linked immunosorbent assay (ELISA) using the Mouse IL-17 ELISA Kit (CSB-E04608m, Cusabio, China) and Mouse IL-4 ELISA Kit (CSB-E04634m, Cusabio, China). All experiments were performed according to the manufacturers’ instructions.

### 2.12 Chromatin immunoprecipitation assay

Chromatin immunoprecipitation (ChIP) assay was conducted by using the ChIP assay kit (Millipore, Cat. NO. 17–371) according to the manufacturer’s instructions. First, 37% formaldehyde was added to the medium to fix the cross-linking of protein and DNA. The cells were then collected and washed three times in cold PBS, centrifuged and resuspended in SDS lysis buffer. The cell lysates were then sonicated to crosslink DNA fragments between 200 and 1000 base pairs in length. To obtain input, 10 μL of supernatant was prewashed with protein G agarose and centrifuged at 4000 × *g* for 1 min, and then the supernatant was stored at 4°C. Next, we added anti-MBD2 antibody and rabbit IgG to the remaining supernatant and incubated it overnight at 4°C with rotation to pull down the immunoprecipitation (IP) products. To collect antibody/antigen/DNA complexes, 60 μL protein G agarose was added to each IP and incubated for 1 h at 4°C by rotation. Then, 100 μL of elution buffer was added to each tube containing the antibody/agarose complex (including the input) for elution; this was incubated at room temperature for 15 min. Subsequently, crosslinking of the eluted protein/DNA complex was reversed by the addition of 5 M NaCl and incubated overnight at 65°C. Then 1 ml of binding reagent A was added, and the DNA flow-through was collected with a spin filter, separated, and purified. The isolated DNA was detected by ChIP-PCR using the following primers: F, 5′-ACG​GCG​GCA​GCG​GAG​T-3′ and R, 5′-AGG​TCG​ATG​TCG​TCC​AGG​CT-3′. The value of quantitative PCR was normalized with input DNA for comparison.

### 2.13 Statistics analysis

All experiments were performed at least 3 times, and all data were expressed as mean ± standard deviation (M ± SD). The differences among different groups were analyzed by one-way analysis of variance (ANOVA) or Kruskal–Wallis test followed by Dunn’s multiple comparisons test. All statistical analyses and graph generation were performed using GraphPad Prism 8.0.1 software (GraphPad Software Inc.). A *p*-value < 0.05 indicated a statistically significant difference.

## 3 Results

### 3.1 A Th17-dominant asthma mouse model was established

**FIGURE 1:**
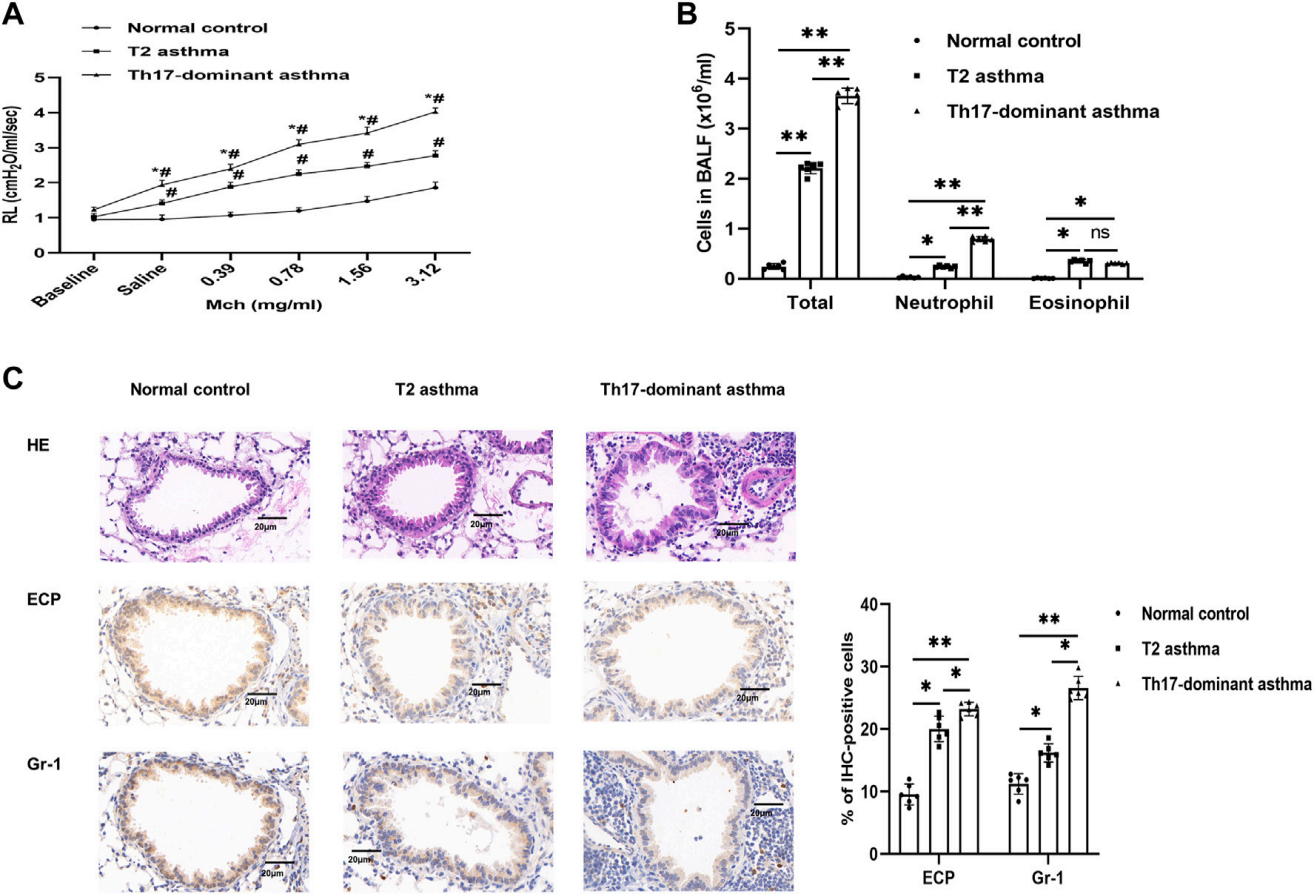
A Th17-dominant asthma mouse model was established **(A)** Lung resistance in the normal control, T2 asthma, and Th17-dominant asthma groups. #*p* < 0.05 as compared to the normal control group. *p < 0.05 as compared to the T2 asthma group. **(B)** Three groups of BALF cells: total, neutrophil, and eosinophil cells **(C)** Lung tissues of the three groups were stained with H&E and immunohistochemical staining performed with [neutrophil-specific antibody (anti-Gr-1) and eosinophil antibody (anti-ECP)]. Scale bar, 20 μm *p < 0.05. **p < 0.01. IHC, immunohistochemistry.

To assess whether Th17-dominant asthma was established, airway resistance and BALF cells induced by Mch were measured on day 21. Compared to the normal control and T2 asthma mice, mice with Th17-dominant asthma had higher baseline RL. After Mch challenge, the RL of these mice increased significantly, especially in the mice with Th17-dominant asthma (Figure 1A). In BALF of the three groups, we found that the mice with Th17-dominant asthma had the highest total cell counts and neutrophil counts, while the mice with T2 asthma had the highest eosinophil counts (Figure 1B). Histological analysis of the lungs showed significantly increased peribronchial inflammatory cell infiltration in the mice with Th17-dominant asthma compared to the normal control and T2 asthmatic mice (Figure 1C). Immunohistochemistry of Gr-1 (neutrophil-specific antibody) confirmed that neutrophil infiltration in the lung was significantly increased in the Th17-dominant asthma group compared to the T2 asthma group. ECP (eosinophil-specific antibody) immunohistochemistry indicated that eosinophil infiltration was higher in both the Th17-dominant asthma and T2 asthma groups than that in the normal control group, but increased significantly in the T2 asthma group (Figure 1C).

### 3.2 Th17-dominant asthma mediated by Th17 cells

**FIGURE 2:**
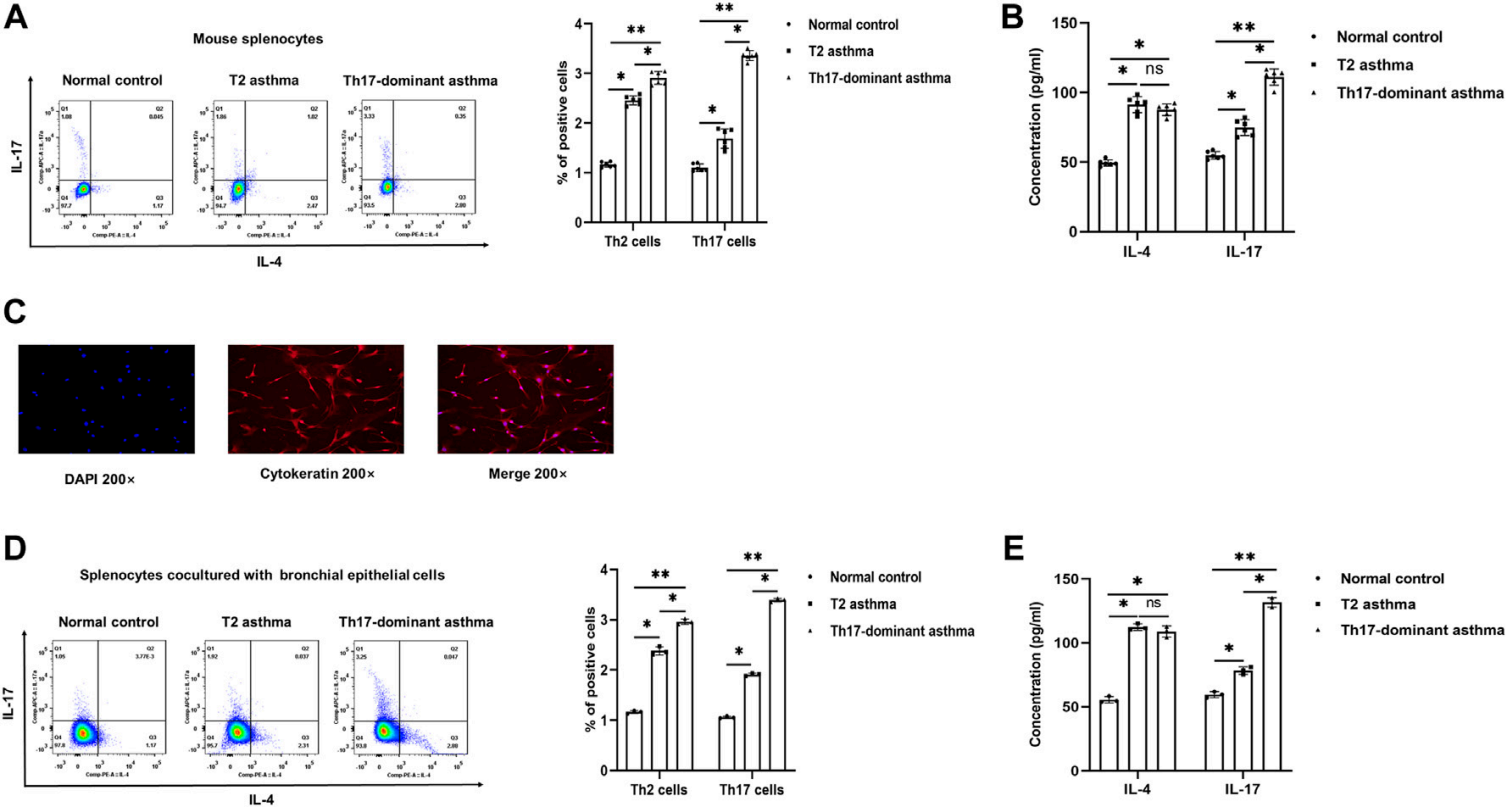
Th17-dominant asthma mediated by Th17 cells **(A)** Th17 and Th2 cells were detected by flow cytometry in splenocytes. **(B)** Serum levels of IL-17 and IL-4 were detected by ELISA **(C)** The positive rate of cytokeratin in BECs was determined by immunofluorescence. **(D and E)** After BECs were stimulated with 100 μg/ml HDM, 100 μg/ml HDM +100 ng/ml LPS or PBS for 24 h and cocultured with CD4^+^ T cells for 24 h, the expression of Th2 and Th17 cells was detected by flow cytometry, and the levels of IL-17 and IL-4 in the cell supernatant were detected by ELISA. *p < 0.05. **p < 0.01.

IL-4 and IL-17 are representative cytokines of Th2 and Th17 cells, respectively. To evaluate whether Th17-dominant asthma is mainly driven by Th17 cells, Th2 and Th17 cells in mouse spleen CD4^+^ T cells were detected by flow cytometry. Compared to the normal and T2 asthma groups, both Th2 and Th17 cells were increased in the Th17-dominant asthma group, especially Th17 cells, while Th2 cells were mainly increased in the T2 asthma group (Figure 2A). The blood serum IL-17 level in the Th17-dominant asthma group was significantly higher than that in the T2 asthma group, while the serum IL-4 level was significantly higher in the T2 asthma group (Figure 2B).

BECs could be used as antigen presenting cells to initiate an immune response. After isolation, immunofluorescence identification results showed that the rate of cytokeratin positive cell population of BECs in mice was more than 90% (Figure 2C). Then, BECs were treated with HDM + LPS, HDM or PBS for 24 h and cocultured with mouse spleen CD4^+^ T cells. Flow cytometry was used to detect the differentiation of Th2 and Th17 cells to evaluate the establishment of cellular asthma model. The results showed that compared to the PBS and HDM groups, Th2 and particularly Th17 cells were increased in the HDM + LPS group, while Th2 cells were mainly increased in the HDM group (Figure 2D). The level of IL-17 in the cell supernatant was significantly higher in the Th17-dominant asthma group than in the T2 asthma group, while that of IL-4 was significantly higher in cell supernatant of the T2 asthma group (Figure 2E). These results suggested that Th17-dominant asthma was mainly mediated by Th17 cells, while Th2 cells were mainly mediated by T2 asthma. In addition, BECs could be used as APCs to initiate the immune response and mainly induced Th17-dominant asthma in the HDM + LPS-exposed group and T2 asthma in the HDM-exposed group.

### 3.3 Expression of MBD2 and MINK1 in Th17-dominant asthma

**FIGURE 3:**
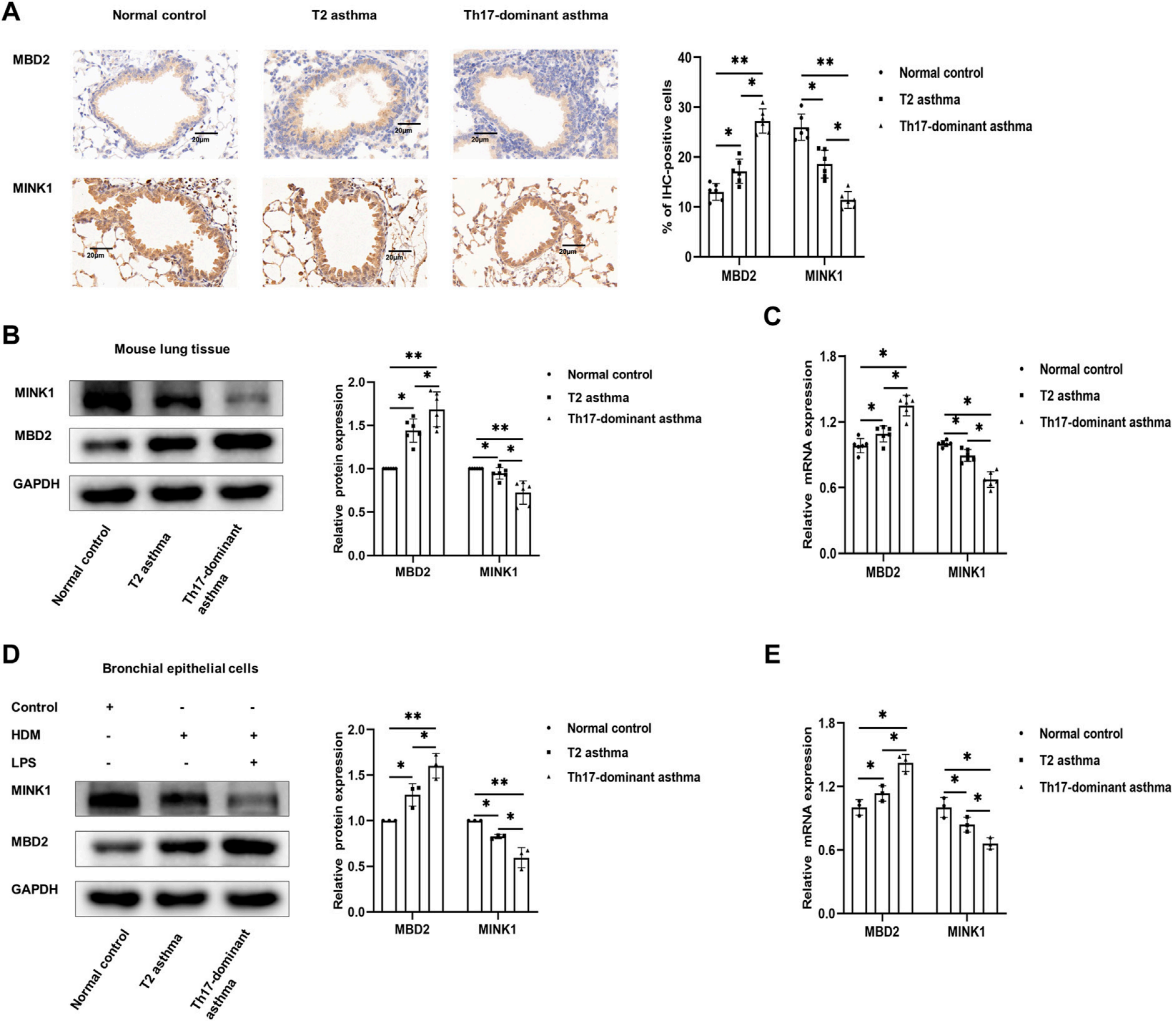
Expression of MBD2 and MINK1 in Th17-dominant asthma **(A)** Lung tissues of each group were stained for anti-MINK1 and anti-MBD2. Scale bar, 20 µm. **(B and C)** The expression of MINK1 and MBD2 protein and mRNA in lung tissues of each group was detected by western blot and qRT-PCR. **(D and E)** The expression of MINK1 and MBD2 protein and mRNA in BECs of each group was detected by western blot and qRT-PCR. *p < 0.05. **p < 0.01. IHC, immunohistochemistry.

It has been reported that MBD2 is significantly increased in neutrophil-dominant asthma and positively regulates Th17 differentiation, while MINK1 negatively regulates Th17 cell differentiation, which has not been reported in Th17 dominant asthma (Fu et al., 2017; Sun et al., 2018). In the present study, the results showed that compared to the asthma group, the histological analyses of lungs in the Th17-dominant asthma group showed significantly increased MBD2 staining and significantly decreased MINK1 staining (Figure 3A). Compared to the T2 asthma group, the expression of MBD2 protein and mRNA in lung tissues and BECs from the Th17-dominant asthma group was significantly increased, while the corresponding expression of MINK1 mRNA and protein was significantly impaired (Figures 3B–D,F). These data suggested that MBD2 and MINK1 expression were associated with asthma phenotype and severity, and that both were involved in the pathogenesis of Th17-dominant asthma.

### 3.4 MBD2 is needed to maintain MINK1 silencing

**FIGURE 4:**
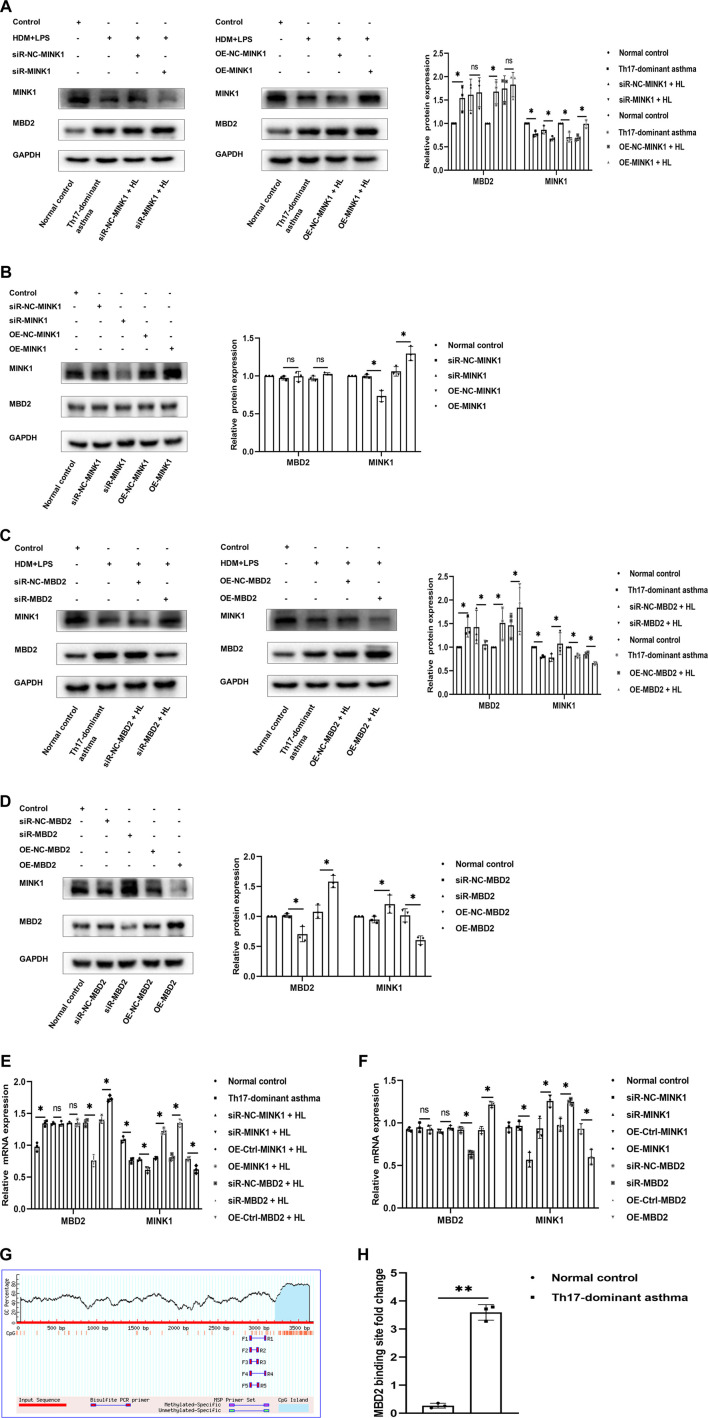
MBD2 is needed to maintain MINK1 silencing **(A, B, E, and F)** Western blot and qRT-PCR were used to verify the transfection of the MINK1 gene with or without HDM + LPS exposure. (A, B, E, and **(F)** Western blot and qRT-PCR were used to detect the expression of MBD2 protein and mRNA in MINK1 gene silencing or overexpression with or without HDM + LPS exposure (C, D, E, and **(F)** Western blot and qRT-PCR were used to verify the transfection of the MBD2 gene with or without HDM + LPS exposure. **(C,D,E and F)** Western blot and qRT-PCR were used to detect the expression of MINK1 protein and mRNA in MBD2 gene silencing or overexpression with or without HDM + LPS exposure **(G)** The patterns of CpG islands of the MINK1 promoter and one pair of the primers were predicted by MethPrimer Promoter 2.0 software. **(H)** Quantitative PCR results of MBD2-bound MINK1 promoter region. *p < 0.05. **p < 0.01. HL, HDM + LPS.

Next, to further understand the molecular mechanism of MBD2 involvement in Th17-dominant asthma, we attempted to determine whether there was any relationship between MBD2 levels and MINK1 expression in BECs exposed or not exposed to HDM + LPS. First, we confirmed by using western blot and qRT-PCR analysis that MINK1 gene silencing or overexpression in BECs with or without HDM + LPS exposure was successful. Then, we detected the expression of MBD2 in each group. There was no significant difference in MBD2 mRNA and protein expression when the MINK1 gene was silenced or overexpressed (Figures 4A,B,E,F). We then used western blot and qRT-PCR analysis to confirm the successful silencing or overexpression of the MBD2 gene in BECs with or without HDM + LPS exposure. Then, we detected the expression of MINK1 in each group. The results showed that the expression of MINK1 mRNA and protein was increased after MBD2 gene silencing compared to the control group. As expected, when the MBD2 gene was overexpressed, MINK1 mRNA and protein expression were significantly reduced compared to the blank and control groups (Figures 4C–F). In addition, we performed a search using the online software MethPrimer (http://www.uroge ne. org/methp Rimer 2/) to analyze the promoter region of MINK1. The prediction of CpG islands is shown in Figure 4G. ChIP assay showed that HDM + LPS exposure significantly increased MBD2 binding to the MINK1 gene promoter region (Figure 4H). These results suggest that MBD2 is involved in maintaining MINK1 silencing.

### 3.5 Th17 cell differentiation under MBD2 and MINK1 gene silencing or overexpression

**FIGURE 5:**
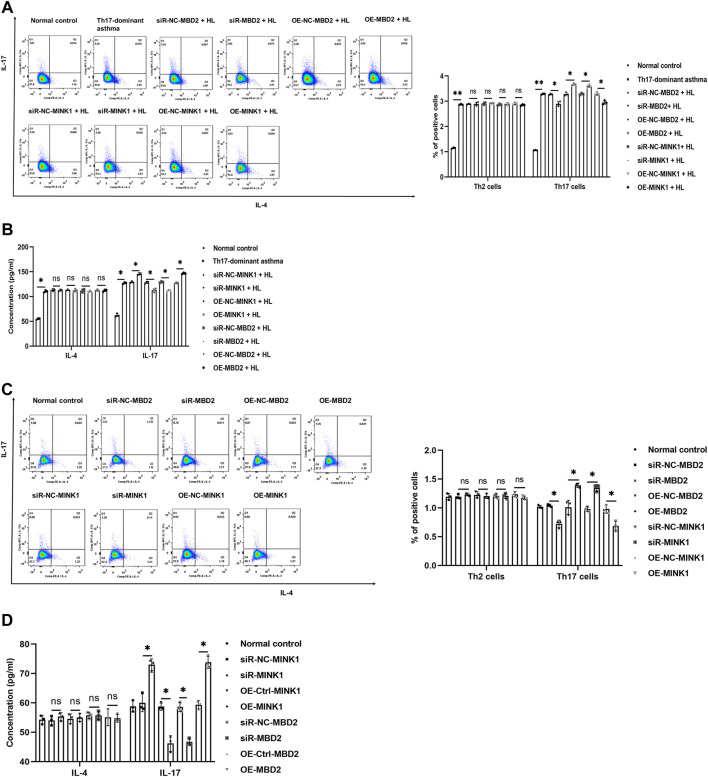
Th17 cell differentiation under MBD2 and MINK1 gene silencing or overexpression **(A and B)** After the MBD2 or MINK1 gene was transfected in BECs with HDM + LPS exposure and cocultured with CD4^+^ T cells for 24 h, the expression of Th2 and Th17 cells was detected by flow cytometry, and the levels of IL-17 and IL-4 in the cell supernatant were detected by ELISA. **(C and D)** After the transfection of the MBD2 or MINK1 gene in BECs without HDM + LPS exposure and cocultured with CD4^+^ T cells for 24 h, the expression of Th2 and Th17 cells was detected by flow cytometry, and the levels of IL-17 and IL-4 in the cell supernatant were detected by ELISA. *p < 0.05. **p < 0.01. HL, HDM + LPS.

Th17-dominant asthma is mainly mediated by Th17 cells, and both MBD2 and MINK1 genes can affect the differentiation of Th17 cells. In order to further understand the regulatory effects of MBD2 and MINK1 on Th17 cells, after silencing or overexpression of MINK1 and MBD2 genes in BECs with or without HDM + LPS exposure, the cells were cocultured with spleen CD4^+^ T cells. Then the differentiation of Th17 cells was analyzed by flow cytometry, and the level of IL-17 was detected by ELISA. The results showed that Th17 cells and the cytokine IL-17 expressed the same trend when MBD2 was silenced or overexpressed. However, when MINK1 was silenced or overexpressed, Th17 cell differentiation and IL-17 showed the opposite trend, increasing or decreasing, respectively. Together, these data indicated that MBD2 induced Th17 cell differentiation by maintaining MINK1 silencing, thereby contributing to the development of Th17-dominant asthma (Figures 5A–D).

## 4 Discussion

Asthma is a highly heterogeneous airway inflammatory disease. Some patients with asthma have a poor response to glucocorticoid therapy and are prone to developing severe asthma. This type of glucocorticoid-resistant severe asthma has been reported to be associated with Th17 cell-mediated neutrophil inflammation (Manni et al., 2016). In order to better study the pathogenesis of severe asthma, it is necessary to establish a Th17-dominant asthma model. OVA is the classic allergen to induce T2 asthma, and HDM can also induce allergic asthma mainly with Th2 cells (Yasuda et al., 2020). LPS is used as an auxiliary agent in the induction of an asthma model, which is associated with increased severity of asthma. In addition to inhibiting Th2 cell differentiation and eosinophil inflammation, the addition of LPS during HDM induction also transformed to neutrophil inflammation (Daan de Boer et al., 2013; Jia et al., 2017). According to previous reports, in this study, 100 μg OVA +100 μg HDM +15 μg LPS was used to successfully establish a Th17-dominant asthma mouse model. HDM + LPS + OVA-exposed mice with Th17-dominant asthma had greater AHR, higher total BALF cell and neutrophil counts, more lung inflammatory cells, higher IL-17 levels and Th17 cells in splenocytes compared to OVA-exposed asthma mice. At the same time, higher differentiation of Th17 cells and IL-17 levels were found in the Th17-dominant asthma induced by HDM + LPS exposure in BECs compared to HDM-induced T2 asthma. These results suggest that the dominant differentiation of Th17 cells and recruitment of neutrophils are the main manifestations of Th17-dominant asthma, thus promoting the development of asthma. In addition, BECs could be used as APCs to initiate an immune response. Liu et al. found that bombesin receptor subtype-3 (BRS-3) has a protective effect on BECs with oxidative damage, and the activation of BRS-3 could increase the uptake of antigen by BECs and T cell proliferation (Liu et al., 2018).

As a reader of DNA methylation, MBD2 plays a role in regulating Th17 cell differentiation through the T-bet/Hlx axis (Zhong et al., 2014). Considering the role of Th17 cells in glucocorticoid-insensitive asthma, MBD2 may be involved in the pathogenesis of Th17-dominant asthma. In this study, MBD2 was significantly increased in lung tissues and BECs in the Th17-dominant asthma groups compared to that in T2 asthma groups. To verify that MBD2 is involved in Th17-dominant asthma, we performed an *in vitro* study on BECs. The MBD2 gene was silenced or overexpressed in BECs with or without HDM + LPS-exposed, and Th17 cells and the cytokine IL-17 showed the same changes with MBD2 gene silencing or overexpression. These results suggested that MBD2 is involved in the development of severe asthma by influencing the differentiation of Th17 cells. Xu et al. found that MBD2 could affect the differentiation of Th17 cells by regulating the key transcription factor retinoid-related orphan nuclear receptor γt of Th17 cells (Xu et al., 2018). MBD2 is widely expressed and plays a role in inflammatory pathogenesis, resulting in transcriptional silencing by interacting with nucleosome remodeling and histone deacetylase complexes as inhibitors (Wood et al., 2016). It was found that elevated MBD2 mRNA levels in CD4^+^ T cells of systemic lupus erythematosus (SLE) patients were positively correlated with the SLE disease activity index (Qin et al., 2013).

MINK1, a serine-threonine kinase, is involved in gene transcription, the inflammatory response, and T cell differentiation. The nucleotide-binding domain, leucine-rich-repeat containing family, pyrin domain-containing 3 (NLRP3) inflammasome plays a pathogenic role in inflammatory diseases, and MINK1 positively regulates NLRP3 inflammasome. In the mouse model of acute sepsis, MINK1 deficiency reduces the NLRP3 activation and inhibits the inflammatory response (Zhu et al., 2021). MINK1 can also affect tumor immunity. Studies have found that the Hippo pathway kinase LATS1/2 in tumor cells can affect tumor growth, and MINK1 is involved in the expression of Hippo pathway kinase. Combined deletion of MINK1 and MST1/2 could inhibit the activation of LATS1/2, improve tumor immunogenicity, and inhibit tumor growth (Meng et al., 2015; Moroishi et al., 2016). It has been reported that MINK1 negatively regulates Th17 cell differentiation and has a protective effect on EAE (Fu et al., 2017). In this study, we found that MINK1 was significantly reduced in lung tissues and BECs in the Th17-dominant asthma groups compared to that in T2 asthma groups. We also carried out *in vitro* experiments on the MINK1 gene and found that Th17 cell differentiation and the IL-17 level increased when the MINK1 gene was silenced but decreased when the MINK1 gene was overexpressed, demonstrating the inverse expression trend. At the same time, there was no significant difference in MBD2 expression when the MINK1 gene was silenced or overexpressed. *In vitro* experiments, we further found that the expression of MINK1 was increased when the MBD2 gene was silenced and decreased when the MBD2 gene was overexpressed, confirming that MBD2 could negatively regulate the expression of MINK1. In addition, ChIP assay revealed that MBD2 could directly bind to CpG islands in the MINK1 promoter region. These results suggested that MINK1 plays a protective role in asthma by negatively regulating Th17 cell differentiation and is negatively regulated by MBD2. In addition, these results indicated that Th17 cells are not directly downstream of MBD2, and MBD2 regulates Th17 cell differentiation through negative regulation of MINK1 expression, thus mediating the onset of Th17-dominant asthma.

## 5 Conclusion

In conclusion, the data from this study demonstrated that MBD2-mediated Th17 cell differentiation is associated with reduced MINK1 expression in Th17-dominant asthma. Our findings have revealed new roles for MBD2 and MINK1 and provide new insights into epigenetic regulation of Th17-dominant asthma, which is dominated by neutrophils and Th17 cells. This study could lead to new therapeutic targets for patients with severe asthma.

## Data Availability

The original contributions presented in the study are included in the article/supplementary material, further inquiries can be directed to the corresponding authors.
